# Understanding the association between self-reported poor oral health and exposure to adverse childhood experiences: a retrospective study

**DOI:** 10.1186/s12903-020-1028-6

**Published:** 2020-02-14

**Authors:** Kat Ford, Paul Brocklehurst, Karen Hughes, Catherine A. Sharp, Mark A. Bellis

**Affiliations:** 1 Public Health Collaborating Unit, School of Health Sciences, College of Human Sciences, Bangor University, Wrexham, LL13 7YP Wales; 2 NWORTH, School of Health Sciences, College of Human Sciences, Bangor University, Gwynedd, LL57 2UW Wales; 3 Policy and International Health Directorate, World Health Organization Collaborating Centre on Investment for Health and Wellbeing, Public Health Wales, Wrexham, LL13 7YP Wales

**Keywords:** Dental health, Oral health, Adverse childhood experiences, Child maltreatment, child abuse, tooth loss, Public health

## Abstract

**Background:**

Adverse childhood experiences, including physical, sexual or emotional abuse, can have detrimental impacts on child and adult health. However, little research has explored the impact that such early life experiences have on oral health. This study examines whether experiencing adverse childhood experiences before the age of 18 years is associated with self-reported poor dental health in later life.

**Methods:**

Using stratified random probability sampling, a household survey (*N* = 5307; age range 18–69 years) was conducted in the South of England (Hertfordshire, Luton and Northamptonshire). Data were collected at participants’ homes using face-to-face interviews. Measures included exposure to nine adverse childhood experiences, and two dental outcomes: tooth loss (> 8 teeth lost due to dental caries or damage) and missing or filled teeth (direct or indirect restorations; > 12 missing or filled teeth).

**Results:**

Strong associations were found between exposure to childhood adversity and poor dental health. The prevalence of tooth loss was significantly higher (8.3%) in those with 4+ adverse childhood experiences compared to those who had experienced none (5.0%; *p* < 0.05). A similar relationship was found for levels of missing or filled teeth (13.4%, 4+ adverse childhood experiences; 8.1%, none; *p* < 0.001). Exposure to 4+ adverse childhood experiences was associated with a higher level of tooth loss and restorations at any age, compared to individuals who had not experienced adversity. Demographically adjusted means for tooth loss increased with adverse childhood experience count in all age groups, rising from 1.0% (18–29 years) and 13.0% (60–69 years) in those with none, to 3.0% and 26.0%, respectively in those reporting 4+.

**Conclusions:**

Exposure to childhood adversity could be an important predictive factor for poor dental health. As oral health is an important part of a child’s overall health status, approaches that seek to improve dental health across the life-course should start with safe and nurturing childhoods free from abuse and neglect. Given the growing role that dental professionals have in identifying violence and abuse, it seems appropriate to raise awareness in the field of dentistry of the potential for individuals to have suffered adverse childhood experiences, and the mechanisms linking childhood adversity to poor dental health.

## Background

The term “adverse childhood experiences” (ACEs) is used to define exposure to abuse or household dysfunction before the age of 18 years. ACEs include suffering emotional, physical or sexual abuse, or growing up in a household where domestic violence, alcohol or drug misuse, parental incarceration or separation, or mental illness is present. Experiencing trauma or chronic stress imposed by ACEs, has been found to detrimentally impact on the development of nervous, endocrine, and immune systems, altering brain development, and resulting in greater allostatic load [1–4]. A considerable body of international evidence has shown the long-term impacts from ACE exposure, such as: an augmented propensity for health-harming behaviours (for example, poor diets and smoking [5, 6]); antisocial behaviour [7]; development of poor childhood and adult mental health [8, 9]; and greater morbidity and mortality [10, 11]. Furthermore, research is starting to explore the implications of experiencing ACEs on oral health [12–14].

Exposure to physical abuse can directly cause injury to teeth, and studies have found that child maltreatment is associated with chronic oral disease and early childhood caries [15, 16]. Individuals who have suffered sexual abuse are more likely to have dental fear and are less likely to attend dental practices [17–20]. Recent studies in England and Wales have shown that ACEs are associated with poor diets, with individuals who have experienced four or more (4+) ACEs being two times more likely to report a poor diet than those who experienced no ACEs [5, 21]. As an unhealthy diet is a risk factor for oral disease [22], it is possible that ACEs could be associated with dental caries and poor oral health. Children are also likely to be reliant upon their parents or carers for their diet and maintenance of their oral health [22], thus, growing up in a dysfunctional household where ACEs are present may negatively affect their dental outcomes. Despite this, only a few studies have measured associations between ACEs and dental outcomes. Studies of children in the USA have found exposure to ACEs to negatively impact on oral health-related quality of life [12] and increase the likelihood of poor oral health and dental caries [13]. Retrospective studies in adults in Japan and the USA, have identified significant associations between exposure to ACEs and having fewer remaining teeth [14] and inadequate dental care [23]. No studies to date have examined associations between ACEs and oral health in other countries. To our knowledge, this is the first study to explore this relationship in the UK, and to examine the potential association of ACEs with the prevalence of missing or filled teeth. Nevertheless, ACEs are reasonably common, with a review finding that, on average, half of all study samples report exposure to at least one ACE [6]. Consequently, it is important that the associations between ACEs and poor oral health are better understood if we seek to mitigate any long-term harms associated with exposure to childhood trauma.

### Aims

We aimed to investigate any association between ACEs and self-reported poor oral health in later life, as measured by (a) having teeth removed or lost due to dental caries or damage and (b) having teeth that had been directly restored (i.e. restorations inside the mouth such as a filling) or indirectly restored (i.e. restorations made outside of the mouth and then inserted such as a crown or a cap), among adults in the South of England.

## Methods

From June to September 2015, a cross-sectional household survey was completed within three geographical regions in the South of England (Hertfordshire, Luton and Northamptonshire). Based on ACE prevalence in other surveys [5] a target sample size of 5588 was set to enable sub-regional modelling of ACE prevalence within local authorities (details available online [24]). In order to ensure our sample was representative of the deprivation profile across the target population, we used a stratified random sampling technique with stratification at the Lower Super Output Area (LSOA; geographical areas with approximately 1500 residents) level. LSOAs were categorised into urban/rural categories (urban city and town; urban major conurbation; rural town and fringe; rural village and dispersed), population ethnicity profile (four ethnic categories: low Black, low Asian; low Black, high Asian; high Black, low Asian; high Black, high Asian) and deprivation quintiles (1 = least deprived to 5 = most deprived). For deprivation, LSOAs were categorised using the English Index of Multiple Deprivation (IMD) [25]. The IMD is a standardised composite measure (combining data on access to services, education, employment, income, health, crime and the physical environment) routinely used to compare deprivation between small discrete localities. Although LSOA level data are an ecological measure, they have been used routinely to assign characteristics to residents within an area that are not available on each individual [26]. Consequently, we undertook a stratified random sampling approach at LSOA level [27] with stratification by deprivation, urban/rural categorisation and ethnicity. LSOAs were randomly selected to match the overall proportions of LSOAs within these categories across regions. Within each sampled LSOA (*n* = 278), households were randomly selected for inclusion using the postcode address file (for more information on sampling see [24]).

### Measures

All measures were self-reported and are provided in the Additional file 1. Standardised ACE survey questions from the US Centers for Disease Control and Prevention short ACE tool [28] were used to measure exposure (before 18 years of age) to childhood abuse and family dysfunction (Table 1). Data on dental outcomes were collected using two questions, preceded with the statement ‘*Adults usually have up to 32 teeth, including 4 wisdom teeth* … ’ (1) *Roughly how many adult teeth have you lost or had taken out due to decay or damage?*; and, (2) *Roughly how many of your (remaining) teeth have fillings or crowns/caps? (This does not include veneers)*. Participant socio-demographic information collected included: age (18–29; 30–39; 40–49; 50–59; and 60–69 years), gender (male; female), ethnicity (self-defined using UK census categories) and residential deprivation quintile.

**Table 1:** Full questions used in survey to measure ACEs and coding

ACE questions. All ACE questions were preceded by the statement “While you were growing up, before the age of 18...”		
ACE	Question	Qualifying response
*Physical abuse*	How often did a parent or adult in your home ever hit, beat, kick, or physically hurt you in any way? This does not include gentle smacking for punishment?	Once or more than once
*Verbal abuse*	How often did a parent or adult in your home ever swear at you, insult you, or put you down?	More than once
*Sexual abuse*	How often did anyone at least 5 years older than you (including adults) ever touch you sexually?	Once or more than once to any of the questions
	How often did anyone at least 5 years older than you (including adults) try to make you touch them sexually?	
	How often did anyone at least 5 years older than you (including adults) force you to have any type of sexual intercourse (oral, anal, or vaginal)?	
*Parental separation*	Were your parents ever separated or divorced?	Yes
*Domestic violence*	How often did your parents or adults in your home ever slap, hit, kick, punch, or beat each other up?	Once or more than once
*Mental illness*	Did you live with anyone who was depressed, mentally ill, or suicidal?	Yes
*Alcohol abuse*	Did you live with anyone who was a problem drinker or alcoholic?	Yes
*Drug abuse*	Did you live with anyone who used illegal street drugs or who abused prescription medications?	Yes
*Incarceration*	Did you live with anyone who served time or was sentenced to serve time in a prison or young offenders’ institution?	Yes

### Procedure

A market research company was commissioned by Luton Borough Council to undertake the fieldwork. Selected households were sent letters outlining the study methodology, information on how to opt out of the survey and contact details for the research team. Households which did not opt out were visited by a trained interviewer (Monday-Sunday, 9 am-8 pm; multiple visits [≤5] were made to selected households to recruit participants). Interviewers could arrange to call back at a time/date more suitable to potential participants or for the interview to be conducted using an interpreter. The study inclusion criteria were: resident in a selected LSOA, aged 18–69 years, and cognitively able to participate. One resident from each household was invited to take part in the survey. Where more than one eligible resident was present, the “next birthday” rule was used to randomly select the participant.

On contact, the interviewer verbally provided potential participants with further information on the study including: its purpose and voluntary, anonymous and confidential nature; their right to withdraw at any time; and that participation or a decline to participate would not affect their health treatment or service provision. All participants were given the opportunity to ask questions and provided verbal informed consent before proceeding with the survey. Questionnaires were completed using CAPI (computer-assisted personal interviewing), with CASI (computer-assisted self-interviewing) utilised for more sensitive questions (e.g. ACE questions). Respondents could opt to be interviewed in a range of languages including Balochi, Bengali, French, Gujarati, Hindi, Marathi, Pashto, Polish, Punjabi, Saraiki Sindhi, Spanish and Urdu. Following survey completion, participants were provided with a thank you leaflet including contact details for the research team and information on national and local help and support services. No personal identifiable data were collected from participants at any stage during the recruitment process or interview.

After receiving the letter advertising the study, 1298 households opted out. During the study period, contact was made with 9929 residents, 1149 (11.6%) of whom were not eligible to participate, 3101 (31.2%) declined participation and in 56 instances language not could not be accommodated. A total of 5623 residents completed the questionnaire, resulting in a participation rate of 64.0% of eligible occupied households (55.8% of all households when accounting for opt out at letter stage). For the study, cases without full socio-demographics and/or ACE questions (*n* = 169), or dental outcomes (*n* = 147) were removed from the sample, resulting in a sample size of 5307 (drawn from 278 LSOAs, mean number of respondents per LSOA = 19, range 2–40).

### Analyses

Statistical analyses were completed using SPSS v24.

In line with previous ACE studies [6], responses to ACE items were used to calculate an individual’s total ACE score (total of nine ACEs), which were used to categorise an individual’s ACE count (0, 1, 2–3 and 4+). The total self-reported number of teeth lost/removed due to dental caries or damage (tooth loss), and the total self-reported number of teeth that had direct or indirect restorations, were summed to create the variable missing or filled teeth. Responses to both outcomes of interest (tooth loss; missing or filled teeth) were dichotomised to indicate poor levels of each outcome. We categorised high scores for the total number of teeth lost, or the summed variable missing or filled teeth as > 1 standard deviation (SD) above the mean (tooth loss, mean 2.8, SD 5.2, high > 8; missing or filled teeth, mean 5.8, SD 6.3, high > 12) to indicate poor dental outcomes. Ethnicity was re-categorised for the purposes of analysis (White, Asian and other).

Analyses used cross-tabulations and chi-square tests to initially examine bivariate associations between ACEs, socio-demographic characteristics and dental outcomes. Binary logistic regression was employed to examine the independent relationships between ACEs and the dental outcomes of interest. Due to research highlighting the associations between ACEs and socio-demographics (e.g. higher ACE prevalence in more deprived communities, lower prevalence in those of Asian ethnicity [5]), we adjusted the models for participant demographics (i.e. age, gender and ethnicity) at an individual level and deprivation was adjusted for at an LSOA level in order to account for socio-economic factors included in the IMD. Finally, fitted binary logistic regression models were used to generate the estimated adjusted proportions with dental outcomes for individuals in different ACE categories and age groups, while adjusting to keep the effects of deprivation and ethnicity constant. Model estimates were calculated using the generalized linear models function in SPSS (model type binary logistic regression, estimated marginal means [29]).

### Ethical approval

Ethical approval was obtained for the study from the Liverpool John Moores University Research Ethics Committee (reference 14/EHC/007). Interviewers followed the Market Research Society Code of Conduct and adhered to the Declaration of Helsinki.

## Results

The demographic breakdown of the sample is shown in Table 2. More than one in 20 (5.5%; *n* = 293) participants were categorised as having high levels of missing teeth (> 8), and nearly one in 10 (9.5%; *n* = 503) reported high levels of missing or filled teeth (> 12; Table 2). Less than half of all participants reported having suffered at least one ACE (41.9%), with 8.2% reporting exposure to 4+ ACEs. A higher ACE prevalence (4+ ACEs) was associated with being female (9.4% versus male, 6.6%; *p* < 0.05), of a younger age (18–29 years 10.7%, compared to 60–69 years, 4.2%; *p* < 0.001), of White ethnicity (8.9% compared to Asian, 3.9% and other, 7.5%; *p* < 0.001) and resident in more deprived areas (11.6% most deprived, 7.9% least deprived; *p* < 0.05).

**Table 2:** Prevalence of high tooth loss and missing or filled teeth by socio-demographics and ACE count

		All	% > 8 teeth lost	% > 12 missing or filled teeth
All		5307	5.5	9.5
Gender	Male	44.8	6.3	10.4
	Female	55.2	4.9	8.7
	*X*^2^		5.083	4.502
	P		0.024	0.034
Age group	18–29	20.8	2.2	2.3
(years)	30–39	22.7	1.8	3.3
	40–49	20.7	2.0	4.9
	50–59	16.7	5.5	12.8
	60–69	19.1	17.4	26.7
	*X*^2^		354.663	507.557
	P		< 0.001	< 0.001
Ethnicity	White	81.0	6.3	11.0
	Asian	12.7	1.8	2.8
	Other	6.3	3.6	3.3
	*X*^2^		24.878	61.242
	P		< 0.001	< 0.001
Deprivation	1 (Least deprived)	28.8	4.5	11.0
	2	20.4	4.6	9.0
	3	20.9	5.9	8.3
	4	19.6	5.5	7.2
	5 (Most deprived)	10.3	9.7	12.8
	*X*^2^		23.732	19.736
	P		< 0.001	0.001
ACE count	0	58.1	5.0	8.1
	1	18.3	5.6	11.3
	2–3	15.5	5.8	10.4
	4+	8.2	8.3	13.4
	*X*^2^		8.086	19.131
	P		0.044	< 0.001

Higher levels of tooth loss and missing or filled teeth were associated with age (most prevalent in the 60–69 age group), being male and White ethnicity (Table 2). Both outcomes increased with deprivation, with the prevalence of high tooth loss doubling from 4.5% for participants resident in the least deprived quintile, to 9.7% for the most deprived quintile. Although significant associations with deprivation existed for high levels of missing or filled teeth, the relationship was not linear, with a high prevalence of missing or filled teeth also being found in the least deprived quintile. Strong associations were found between ACEs and both dental outcomes, with the proportion reporting high levels of tooth loss, and missing or filled teeth increasing with ACE count. The prevalence of high tooth loss increased from 5.0% in those with no ACEs to 8.3% in those with 4+ ACEs, for missing or filled teeth this was 8.1 and 13.4%, respectively.

Logistic regression analyses accounting for relationships with socio-demographics (i.e. adjusted for age, gender, ethnicity and deprivation), ACEs remained strongly related to both dental outcomes. Models showed a good fit to observed data (see Additional file 1: Table S1). Adjusted odds ratios (AORs) for high tooth loss and missing or filled teeth reached 2.5 and 2.5, respectively, in those with 4+ ACEs (relative to no ACEs; see Table 3). Relationships between both dental outcomes and ethnicity, deprivation and age remained significant. Gender was no longer significantly related to either dental outcome. Variations in model sensitivity and specificity at different predicted cutoff probabilities for high tooth loss and missing and filled teeth are provided in Additional file 1: Table S2.

**Table 3:** Logistic regression analysis presenting adjusted odds ratios for dental outcomes by ACE count and socio-demographics

		> 8 lost teeth^a^			> 12 missing or filled teeth^b^		
		AOR	95% CIs	P	AOR	95% CIs	P
ACE count	0	Ref			Ref		
	1	1.10	0.79–1.54	0.574	1.50	1.16–1.94	0.002
	2–3	1.31	0.92–1.86	0.133	1.47	1.11–1.94	0.007
	4+	2.45	1.63–3.68	< 0.001	2.52	1.80–3.52	< 0.001
Age group (years)	18–29	Ref			Ref		
	30–39	0.94	0.52–1.70	0.840	1.64	0.98–2.73	0.059
	40–49	1.03	0.57–1.86	0.924	2.39	1.47–3.89	< 0.001
	50–59	2.98	1.80–4.93	< 0.001	6.86	4.38–10.75	< 0.001
	60–69	11.83	7.53–18.58	< 0.001	17.79	11.57–27.36	< 0.001
Ethnicity	White	Ref			Ref		
	Asian	0.34	0.18–0.62	< 0.001	0.34	0.21–0.56	< 0.001
	Other	0.69	0.37–1.28	0.241	0.37	0.20–0.70	0.002
Deprivation	1 (Least deprived)	Ref			Ref		
	2	1.14	0.77–1.68	0.510	0.85	0.65–1.13	0.263
	3	1.55	1.08–2.24	0.018	0.85	0.64–1.13	0.259
	4	1.78	1.22–2.60	0.003	0.86	0.64–1.17	0.340
	5 (Most deprived)	4.13	2.75–6.21	< 0.001	2.18	1.56–3.04	< 0.001

95% CIs: 95% confidence intervals; AOR: adjusted odds ratio; Ref: reference category. Gender was also entered into the model but was not significantly related to either dental outcome. Hosmer and Lemeshow Test: ^a^ X^2^ = 5.244, *P* = 0.731; ^b^ X2 = 7.647, *P* = 0.469

**Fig. 1:**
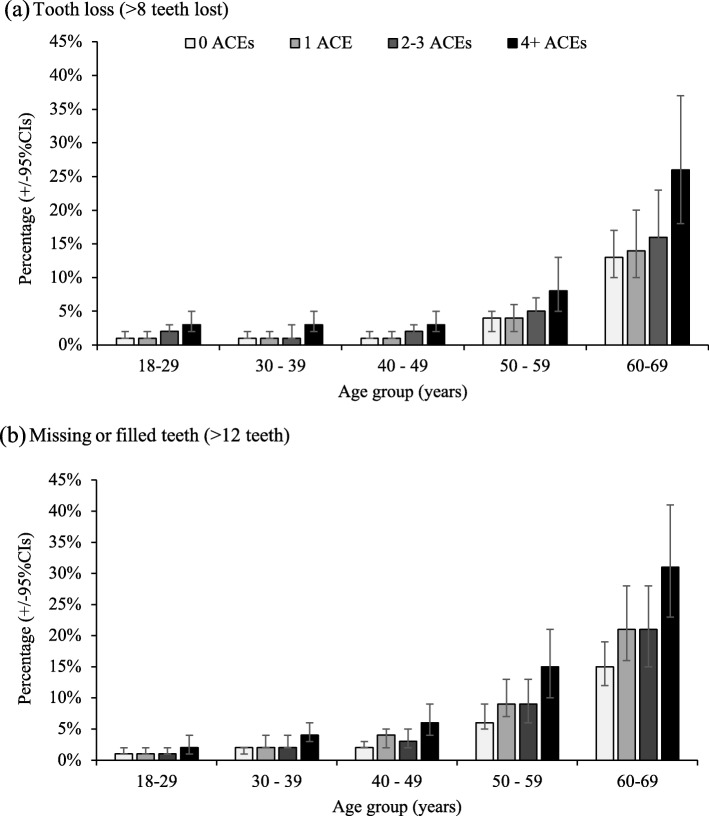
Adjusted mean^$^ percentage of individuals within each dental outcome by age, stratified by ACE count. **a** Tooth loss (> 8 teeth lost). **b** Missing or filled teeth (> 12 teeth). ^$^Adjusted means are calculated using the estimated marginal means function and adjusted through logistic regression modelling for confounding from other variables in the model; here deprivation and ethnicity. 95% CIs: 95% confidence intervals

Adjusted risks of both dental outcomes by ACE count and age were calculated (Fig. 1; unadjusted levels are shown in Additional file 1: Table S3). Modelled levels of tooth loss ranged from 1.0 (18–29 years) to 13.0% (60–69 years) in those without exposure to ACEs, but ranged from 3.0 to 26.0%, respectively in those with 4+ ACEs (adjusting for ethnicity and deprivation; Fig 1a). Equivalent estimates for missing or filled teeth were 1.0 to 15% and 2.0 to 21.0%, respectively (Fig. 1b).

## Discussion

This study identified strong associations between exposure to ACEs and high levels of self-reported tooth loss and restored teeth, suggesting that ACEs could be an important predictive factor for poor oral health. This pattern remained consistent when age, gender, ethnicity and deprivation were accounted for. These findings are consistent with Bright et al. [13] and Matsuyama et al. [14] who found that children exposed to several ACEs are more likely to have poor oral health and that this effect persists into late adulthood.

The clear positive relationship between age and poor oral health identified in these findings was broadly expected, given the deterioration of teeth over time and that older cohorts did not benefit fully from the addition of fluoride into toothpaste. Despite improvements in oral health over the past 40 years [30], having 4+ ACEs at any age was associated with a higher level of tooth loss and restorations, compared with individuals who had experienced no ACEs. For individuals aged 18–29 years, the estimated proportion who had lost > 8 teeth increased from 1.0% in those with no ACEs to 3.0% in those with 4+ ACEs, whilst for the oldest age cohort figures were 13.0% and 26.0%, respectively. These findings suggest that more could be done to prevent dental disease in individuals who are exposed to ACEs.

Overall, the levels of tooth loss and the number of missing or filled teeth reported in this study were similar to those identified within the 2009 UK Adult Dental Health Survey (mean 25.7 teeth amongst dentate adults in England, Wales and Northern Ireland [31]). Our models did not identify significant differences by gender in the relationships between both dental outcomes and exposure to ACEs. In line with other studies, we found inequalities in tooth loss among British adults by deprivation [32], with individuals in the most deprived areas being over four times more likely to have lost > 8 teeth than individuals resident in the least deprived quintiles. Research has shown that ACEs are associated with deprivation, with individuals resident in highly deprived areas more likely to report 4+ ACEs [5]. Adversity is more likely to occur in environments characterised by poverty, violence and low levels of social capital where structural inequalities may exist in the availability of support systems. However, in this study we identified a strong relationship between ACEs and poor self-reported dental outcomes, independent of deprivation.

The mechanisms linking ACE exposure to tooth decay include strong associations between ACEs and other factors that have been found to affect poor oral health, including the adoption of health-harming behaviours such as smoking, poor diet and exposure to violence [5, 6, 21, 24, 33, 34]. In England, individuals exposed to 4+ ACEs have been found to be twice as likely to have a poor diet, three times more likely to be a current smoker, and eight times more likely to have been a victim of violence in the last year, compared to individuals with no ACEs [24]. Other harms associated with ACEs such as poor mental health [9] may also influence dental health, as mental illness may be linked to how much importance individuals place on self-care. Future studies could explore the mediating effects of such behavioural and health outcomes on relationships between ACEs and oral health.

The management of dental disease has a significant cost. Across the European Union, this has been estimated to increase from €54 Billion in 2000 to €93 Billion in 2020; this is greater than the costs for stroke and dementia combined [35]. Equally, the human cost is significant; 34,205 children under ten years of age required treatment in a hospital in England due to tooth decay between April 2016 and March 2017, compared to 19,584 children requiring treatment for asthma [36]. Given the identified relationships between ACEs and poor oral health outcomes and the growing role that dentists are playing in relation to violence prevention and identifying abuse [37, 38], it would seem appropriate to raise awareness among dentists of the potential for individuals to have suffered ACEs and the mechanisms linking ACEs to dental health. This is particularly pertinent given the links between childhood abuse and dental anxiety and dental neglect [19, 20, 39], where it would appear paramount that an understanding of ACEs could help the dental profession engage more with those that have experienced childhood trauma. A recent study found that individuals who have experienced ACEs were in general less trusting of services and less likely to engage with them [9]. Further, as this research was conducted in the general household population, it may underestimate the burden produced by ACEs. There are other population cohorts that are known to exhibit very high levels of ACEs, which have not been captured in this survey. For example, offender populations have been found to have very poor dental health [40] as well as very high exposure to ACEs [41, 42].

The findings from this study are relevant to policy that aims to achieve better oral health for children. Oral health is an important part of a child’s overall health status and a marker of wider health and social care issues [43, 44]. Improving child dental health requires a life-long and whole-systems approach, of which the prevention of ACEs is part of. Life-course approaches to dental health should start with safe and nurturing childhoods free from ACEs, which may help to prevent dental caries and tooth loss alongside other associated issues affecting teeth and overall health, such as smoking and poor diet.

### Limitations

The present findings should be considered in light of several important limitations. The samples were largely demographically representative of the populations being studied and although consistent with other ACE studies [21], the completion rate in this study was 64.0% and bias may have been introduced through self-selection to participate. As no information was recorded on the individuals who declined participation in the study, it is not possible to identify if any association existed between a non-participation and socio-demographics (e.g. we were unable to explore levels of non-participation by deprivation). Consistent with other studies we used LSOA (small geographical areas) as the unit for stratified random sampling. This allowed us to stratify by an established multi-factorial measure of deprivation (IMD). However, using such an ecological measure meant we could not account for any potential variation in deprivation within LSOAs. Future studies could utilise individual level socio-economics for sampling and analysis. However, these are currently not available in England. ACE data were retrospectively collected and subject to recall-bias, preventing any causality between ACEs and dental outcomes being established. Finally, as in other studies [14], dental outcomes were self-reported and it is possible that the reported number of teeth lost or removed were underestimated if individuals failed to recollect these instances. We were also unable to measure how accurately individuals were able to assess the number of teeth with fillings, crowns or caps. Further, we used a standard methodology to categorise our outcomes (one SD below the mean) and the resulting cutoffs were not intended to have clinical significance. Due to limitations on the survey length and the time allowed for completion, only two questions examining dental outcomes were included. These measures were chosen following discussion with local dental public health consultants; however, future studies should seek to explore these associations further.

## Conclusion

This research adds to an increasing literature describing how ACEs can impact life-course health and well-being by examining the associations between exposure to ACEs and dental outcomes, including missing or filled teeth in a large study in the South of England. The present findings indicate that exposure to childhood abuse, and household dysfunction can have life-long implications on dental health. Individuals who were exposed to 4+ ACEs had increased risks of tooth loss and missing or filled teeth at any age. Policies that aim to achieve better oral health across the life-course should start with promoting safe and nurturing childhoods free from exposure to abuse and neglect. Given the increasing focus on the need for dental professionals to identify violence and abuse and vulnerable children, those working in the field of dentistry should be aware of the potential for individuals to have suffered ACEs, and understand the associations linking ACEs to poor dental health.

## Supplementary information

**Additional file 1:** **Tables S1**-**S3.** and Questionnaire Items. Contingency tables for Hosmer and Lemeshow tests (**Table S1**); Logistic regression model classifications at different cut off points for case classification (**Table S2**); Unadjusted proportions with > 8 teeth lost and > 12 missing or filled teeth, by age and ACE count (**Table S3**); Questionnaire items for the ACE and dental health study.

## Data Availability

The datasets used in the current study are available from the corresponding author on reasonable request.

## Abbreviations

ACEs: Adverse childhood experiences
AOR: Adjusted odds ratio
CAPI: Computer-assisted personal interviewing
CASI: Computer-assisted self-interviewing
CIs: Confidence intervals
IMD: Index of multiple deprivation
LSOA: Lower Super Output Area
Ref: reference category

## References

[1] Anda R, Felitti V, Bremner J, Walker JD, Whitfield C, Perry BD (2006). The enduring effects of abuse and related adverse experiences in childhood. Eur Arch Psychiatry Clin Neurosci.

[2] Teicher MH, Samson JA (2016). Annual research review: enduring neurobiological effects of childhood abuse and neglect. J Child Psychol Psychiatry.

[3] Danese A, McEwen BS (2012). Adverse childhood experiences, allostasis, allostatic load, and age-related disease. Physiol Behav.

[4] Pechtel P, Pizzagalli DA (2011). Effects of early life stress on cognitive and affective function: An integrated review of human literature. Psychopharmacology (Berl).

[5] Bellis MA, Hughes K, Leckenby N, Perkins C, Lowey H (2014). National household survey of adverse childhood experiences and their relationship with resilience to health-harming behaviors in England. BMC Med.

[6] Hughes K, Bellis MA, Hardcastle KA, Sethi D, Mikton C, Jones L (2017). The effect of multiple adverse childhood experiences on health: a systematic review and meta-analysis. Lancet Public Health.

[7] Duke NN, Pettingell SL, McMorris BJ, Borowsky IW (2010). Adolescent violence perpetration: associations with multiple types of adverse childhood experiences. Pediatrics..

[8] Hughes K, Lowey H, Quigg Z, Bellis MA (2016). Relationships between adverse childhood experiences and adult mental well-being: results from an English national household survey. BMC Public Health.

[9] Hughes K, Ford K, Davies A, Homolova L, Bellis MA (2018). Sources of resilience and their moderating relationships with harms from adverse childhood experiences. Report 1: mental illness.

[10] Brown DW, Anda RF, Tiemeier H, Felitti VJ, Edwards VJ, Croft JB (2009). Adverse childhood experiences and the risk of premature mortality. Am J Prev Med.

[11] Campbell JA, Walker RJ, Egede LE (2016). Associations between adverse childhood experiences, high-risk behaviors, and morbidity in adulthood. Am J Prev Med.

[12] Kabani F, Lykens K, Tak HJ (2018). Exploring the relationship between adverse childhood experiences and oral health-related quality of life. J Public Health Dent.

[13] Bright MA, Alford SM, Hinojosa MS, Knapp C, Fernandez-Baca DE (2015). Adverse childhood experiences and dental health in children and adolescents. Community Dent Oral Epidemiol.

[14] Matsuyama Y, Fujiwara T, Aida J, Watt RG, Kondo N, Yamamoto T (2016). Experience of childhood abuse and later number of remaining teeth in older Japanese: a life-course study from Japan Gerontological evaluation study project. Community Dent Oral Epidemiol.

[15] Valencia-Rojas N, Lawrence HP, Goodman D (2008). Prevalence of early childhood caries in a population of children with history of maltreatment. J Public Health Dent.

[16] Nicolau B, Marcenes W, Sheiham A (2003). The relationship between traumatic dental injuries and adolescents' development along the life course. Community Dent Oral Epidemiol.

[17] Stalker CA, Russell BDC, Teram E, Schachter CL (2005). Providing dental care to survivors of childhood sexual abuse: treatment considerations for the practitioner. J Am Dent Assoc..

[18] Walker EA, Milgrom PM, Weinstein P, Getz T, Richardson R (1996). Assessing abuse and neglect and dental fear in women. J Am Dent Assoc.

[19] Willumsen T (2001). Dental fear in sexually abused women. Eur J Oral Sci.

[20] Leeners B, Stiller R, Block E, Görres G, Imthurn B, Rath W (2007). Consequences of childhood sexual abuse experiences on dental care. J Psychosom Res.

[21] Bellis MA, Ashton K, Hughes K, Ford K, Bishop J, Paranjothy S (2015). Adverse childhood experiences and their impact on health-harming behaviours in the welsh adult population.

[22] Bhatia SK, Maguire SA, Chadwick BL, Hunter ML, Harris JC, Tempest V (2014). Characteristics of child dental neglect: a systematic review. J Dent.

[23] Crouch C, Radcliff E, Nelson J, Strompolis M, Martin A (2018). The experience of adverse childhood experiences and dental care in childhood. Community Dent Oral Epidemiol.

[24] Ford K, Butler N, Hughes K, Quigg Z, Bellis MA (2016). Adverse childhood experiences (ACEs) in Hertfordshire, Luton and Northamptonshire.

[25] Public Health England. Adjusted IMD 2010 scores for 2011 LSOAs. http://webarchive.nationalarchives.gov.uk/20150505130552/http://www.apho.org.uk/resource/item.aspx?RID=125887. Accessed 11 Feb 2018.

[26] Bellis MA, Lowey H, Hughes K, Deacon L, Stansfield J, Perkins C (2012). Variations in risk and protective factors for life satisfaction and mental wellbeing with deprivation: a cross-sectional study. BMC Public Health.

[27] Lavrakas P (2008). Encyclopedia of Survey Research Methods [Internet].

[28] Centers for Disease Control and Prevention. Behavioral Risk Factor Surveillance System ACE data. https://www.cdc.gov/violenceprevention/childabuseandneglect/acestudy/ace-brfss.html. Accessed 24 Jan 2020.

[29] IBM Knowledge Centre. GLM estimated marginal means. https://www.ibm.com/support/knowledgecenter/SSLVMB_subs/statistics_mainhelp_ddita/spss/common/idh_glm_emmeans.html. Accessed 14 Jan 2020.

[30] Steele JG, Treasure ET, O'Sullivan I, Morris J, Murray JJ (2012). Adult dental health survey 2009: transformations in British oral health 1968-2009. Br Dent J.

[31] Fuller E, Steele J, Watt R, Nuttal N. 1: Oral health and function – a report from the adult dental health survey 2009. The Health and Social Care Information Centre; 2011. http://doc.ukdataservice.ac.uk/doc/6884/mrdoc/pdf/6884theme1_oral_health_and_function.pdf.

[32] Bernabé E, Sheiham A (2014). Tooth loss in the United Kingdom - trends in social inequalities: an age-period-and-cohort analysis. PLoS One.

[33] Csikar J, Kang J, Wyborn C, Dyer TA, Marshman Z, Godson J (2016). The self-reported oral health status and dental attendance of smokers and non-smokers in England. PLoS One.

[34] Millar WJ, Locker D (2007). Smoking and oral health status. J Can Dent Assoc.

[35] Widström E, Eaton KA (2004). Oral healthcare systems in the extended European union. Oral Health Prev Dent.

[36] National Statistics. Hospital admitted patient care activity, 2016-17 - NHS digital. http://digital.nhs.uk/catalogue/PUB30098. Accessed 28 Feb 2018.

[37] Kirkengen AL, Lygre H (2015). Exploring the relationship between childhood adversity and oral health: an anecdotal approach and integrative view. Med Hypotheses.

[38] Rodrigues JLSA, Lima APB, Nagata JY, Rigo L, Franco A, Paranhos LR (2016). Domestic violence against children detected and managed in the routine of dentistry - a systematic review. J Forensic Legal Med.

[39] Brattabø IV, Bjørknes R, Åstrøm AN (2018). Reasons for reported suspicion of child maltreatment and responses from the child welfare - a cross-sectional study of Norwegian public dental health personnel. BMC Oral Health.

[40] Walsh T, Tickle M, Milsom K, Buchanan K, Zoitopoulos L (2008). An investigation of the nature of research into dental health in prisons: a systematic review. Br Dent J.

[41] Ford K, Barton E, Newbury A, Hughes K, Bezeczky Z, Roderick J (2019). Understanding the prevalence of adverse childhood experiences (ACEs) in a male offender population in Wales: the prisoner ACE survey.

[42] Levenson JS, Willis GM, Prescott DS (2014). Adverse childhood experiences in the lives of female sex offenders. Sex Abus.

[43] White S. Health matters: Child dental health - public health matters. https://publichealthmatters.blog.gov.uk/2017/06/14/health-matters-child-dental-health/. Accessed 28 Feb 2018.

[44] Harris JC (2018). The mouth and maltreatment: safeguarding issues in child dental health. Arch Dis Child.

