# Supplementary material for: Understanding the association between self-reported poor oral health and exposure to adverse childhood experiences: a retrospective study
Source: BMC Oral Health. 2020 Feb 14;20:51. doi: 10.1186/s12903-020-1028-6 (PMC7020341; doi:10.1186/s12903-020-1028-6)
Supplement: Supplementary file 1 — Additional file 1: Tables S1-S3. and Questionnaire Items. Contingency tables for Hosmer and Lemeshow tests (Table S1); Logistic regression model classifications at different cut off points for case classification (Table S2); Unadjusted proportions with > 8 teeth lost and > 12 missing or filled teeth, by age and ACE count (Table S3); Questionnaire items for the ACE and dental health study. [file 12903_2020_1028_MOESM1_ESM.docx]

**Table S1: Contingency tables for Hosmer and Lemeshow tests**

|  | >8 teeth lost^a^ | | | | | >12 teeth missing or filled^b^ | | | | |
| --- | --- | --- | --- | --- | --- | --- | --- | --- | --- | --- |
|  | No | | Yes | |  | No | | Yes | |  |
|  | Observed | Expected | Observed | Expected | Total | Observed | Expected | Observed | Expected | Total |
| 1 | 462 | 461.955 | 3 | 3.045 | 465 | 528 | 527.832 | 5 | 5.168 | 533 |
| 2 | 514 | 516.805 | 9 | 6.195 | 523 | 516 | 518.776 | 12 | 9.224 | 528 |
| 3 | 510 | 509.073 | 6 | 6.927 | 516 | 515 | 507.123 | 5 | 12.877 | 520 |
| 4 | 522 | 519.543 | 6 | 8.457 | 528 | 536 | 536.415 | 19 | 18.585 | 555 |
| 5 | 524 | 525.636 | 12 | 10.364 | 536 | 553 | 550.094 | 20 | 22.906 | 573 |
| 6 | 524 | 519.327 | 9 | 13.673 | 533 | 523 | 527.172 | 35 | 30.828 | 558 |
| 7 | 512 | 512.084 | 21 | 20.916 | 533 | 481 | 483.399 | 53 | 50.601 | 534 |
| 8 | 493 | 498.664 | 35 | 29.336 | 528 | 456 | 462.265 | 87 | 80.735 | 543 |
| 9 | 498 | 496.381 | 68 | 69.619 | 566 | 420 | 418.567 | 127 | 128.433 | 547 |
| 10 | 455 | 454.531 | 124 | 124.469 | 579 | 276 | 272.357 | 140 | 143.643 | 416 |

^a^ X^2^ = 5.244, P = 0.731; ^b^ X^2^ = 7.647, P = 0.469.

**Table S2: Logistic regression model classifications at different cut off points for case classification**

| a) Teeth Lost | | | | | | | | b) Teeth Missing | | | | | | |
| --- | --- | --- | --- | --- | --- | --- | --- | --- | --- | --- | --- | --- | --- | --- |
| Cut off | Predicted | Low | High | Low | High |  |  | Cut off | Low | High | Low | High |  |  |
| Probability* | Observed | Low | Low | High | High | Sensitivity | Specificity | Probability* | Low | Low | High | High | Sensitivity | Specificity |
| 0.1 |  | 4123 | 891 | 103 | 190 | 64.85 | 82.23 | 0.1 | 3559 | 1245 | 140 | 363 | 72.17 | 74.08 |
| 0.2 |  | 4825 | 189 | 221 | 72 | 24.57 | 96.23 | 0.2 | 4039 | 765 | 222 | 281 | 55.86 | 84.08 |
| 0.3 |  | 4965 | 49 | 266 | 27 | 9.22 | 99.02 | 0.3 | 4604 | 200 | 385 | 118 | 23.46 | 95.84 |
| 0.4 |  | 5010 | 4 | 287 | 6 | 2.05 | 99.92 | 0.4 | 4749 | 55 | 459 | 44 | 8.75 | 98.86 |
| 0.5 |  | 5014 | 0 | 293 | 0 | 0.00 | 100.00 | 0.5 | 4789 | 15 | 494 | 9 | 1.79 | 99.69 |
| 0.6 |  | 5014 | 0 | 293 | 0 | 0.00 | 100.00 | 0.6 | 4804 | 0 | 501 | 2 | 0.40 | 100.00 |

*Cut off probability is the probability level set within the model for an individual to be classified in the high category.

**Table S3: Unadjusted proportions with >8 teeth lost and >12 missing and filled teeth, by age and ACE count**

|  |  | **>8 teeth lost** | **>12 teeth missing or filled teeth** |
| --- | --- | --- | --- |
| **Age group** | **ACE count** | % | % |
| 18-29 years | 0 | 1.9 | 1.9 |
|  | 1 | 1.4 | 1.8 |
|  | 2-3 | 2.2 | 2.2 |
|  | 4+ | 5.1 | 5.1 |
| 30-39 years | 0 | 0.9 | 1.9 |
|  | 1 | 1.8 | 3.1 |
|  | 2-3 | 2.6 | 4.1 |
|  | 4+ | 7.4 | 12.8 |
| 40-49 years | 0 | 1.6 | 4.1 |
|  | 1 | 1.6 | 3.6 |
|  | 2-3 | 1.8 | 6.1 |
|  | 4+ | 5.6 | 10.2 |
| 50-59 years | 0 | 4.3 | 8.7 |
|  | 1 | 6.6 | 19.1 |
|  | 2-3 | 9.9 | 21.8 |
|  | 4+ | 4.2 | 14.1 |
| 60-69 years | 0 | 16.4 | 23.9 |
|  | 1 | 17.9 | 33.7 |
|  | 2-3 | 16.4 | 23.9 |
|  | 4+ | 33.3 | 45.2 |

ACE = adverse childhood experience

**Childhood Experiences and Health in Hertfordshire, Luton and Northamptonshire**

**Dental study survey items**

**Are you male or female?**

 Male  Female

**How old are you?**

 18-20  21-24  25-29  30-34  35-39

 40-44  45-49  50-54  55-59  60-64

 65-69  70 or over *If 70 or over THANK AND CLOSE - The survey will terminate*

**What is your ethnic group?** Choose one option that best describes your ethnic group or background.

**White**

 White: British (English/Scottish/Welsh/ Northern Irish)

 White: Eastern European

 White: Irish

 White: Gypsy or Irish Traveller

 Other White

**Asian or Asian British**

 Bangladeshi

 Chinese

 Indian

 Pakistani

 Other Asian or Asian British

**Black/African/Caribbean/Black British**

 African

 Caribbean

 Other Black/ African/ Caribbean/ Black British background

**Mixed**

 White and Asian

 White and Black Caribbean

 White and Black African

 Other mixed

**Other**

 Arab

 Other (please state)________

**Roughly how many adult teeth have you lost or had taken out due to decay or damage?**

___________

**Roughly how many of your (remaining) teeth have fillings or crowns/caps? (this does not include veneers)**

___________

**While you were growing up, before the age of 18 years.... Did you live with anyone who was depressed, mentally ill or suicidal?**

 Yes  No  Don’t know

**While you were growing up, before the age of 18 years.... Did you live with anyone who was a problem drinker or alcoholic?**

 Yes  No  Don’t know

**While you were growing up, before the age of 18 years.... Did you live with anyone who used illegal street drugs or who abused prescription medications?**

 Yes  No  Don’t know

**While you were growing up, before the age of 18 years.... Did you live with anyone who served time or was sentenced to serve time in a prison or young offenders institution?**

 Yes  No  Don’t know

**While you were growing up, before the age of 18 years.... Were your parents ever separated or divorced?**

 Yes  No  Don’t know

**While you were growing up, before the age of 18 years.... How often did your parents or adults in your home ever slap, hit, kick, punch or beat each other up?**

 Yes  No  Don’t know

**While you were growing up, before the age of 18 years.... How often did a parent or adult in your home ever hit, beat, kick or physically hurt you in any way? This does not include gentle smacking for punishment.**

 Yes  No  Don’t know

**While you were growing up, before the age of 18 years.... How often did a parent or adult in your home ever swear at you, insult you, or put you down?**

 Yes  No  Don’t know

**While you were growing up, before the age of 18 years.... How often did anyone at least 5 years older than you (including adults) ever touch you sexually?**

 Yes  No  Don’t know

**While you were growing up, before the age of 18 years.... How often did anyone at least 5 years older than you (including adults) try to make you touch them sexually?**

 Yes  No  Don’t know

**While you were growing up, before the age of 18 years.... How often did anyone at least 5 years older than you (including adults) force you to have any type of sexual intercourse (oral, anal or vaginal)?**

 Yes  No  Don’t know
